# Supplementary material for: Granular insights: A wastewater-based machine learning approach for localized COVID-19 hospitalization forecasting
Source: Epidemics. Author manuscript; Available in PMC 2026 Nov 9. (PMC13746233; doi:10.1016/j.epidem.2026.100907)
Supplement: Supplementary file 1 [file NIHMS2215560-supplement-Supplementary_Material.pdf]

Appendix

A. WWTP

Table A.1: Socio-Demographic Characteristics of WWTP Service Areas. Population estimates were based on 2019 U.S. Census ZIP code level data. ZIP codes partially within a WWTP boundary were included in full. (U.S. Census Bureau, 2025)

**WWTP Area**	Richland (Metro)	Richland (ER)	Lexington	York	Charleston (PI*)	Charleston (MP*)
**Sex**						
Male	182888 (49%)	41813 (47%)	114108 (49%)	128368 (49%)	98083 (47%)	47402 (49%)
Female	188787 (51%)	46461 (53%)	117980 (51%)	136152 (51%)	109533 (53%)	50103 (51%)
**Age Group**						
0-19 years	95101 (26%)	21714 (25%)	59554 (26%)	70833 (27%)	40293 (19%)	23249 (24%)
20-44 years	141674 (38%)	30156 (34%)	75252 (32%)	84641 (32%)	80089 (39%)	29383 (30%)
45-64 years	83890 (23%)	21362 (24%)	61923 (27%)	71100 (27%)	52138 (25%)	26995 (28%)
$\geq$ 65 years	51010 (14%)	15042 (17%)	35359 (15%)	37946 (14%)	35096 (17%)	17878 (18%)
**Race or Ethnicity**						
Hispanic	18706 (5%)	5328 (6%)	15611 (7%)	16250 (6%)	7696 (4%)	2165 (2%)
White	176720 (48%)	36574 (41%)	177602 (77%)	190438 (72%)	151656 (73%)	84708 (87%)
Black	161656 (43%)	42864 (49%)	32436 (14%)	50346 (19%)	44279 (21%)	7915 (8%)
Other	33299 (9%)	8836 (10%)	22050 (10%)	23736 (9%)	11681 (6%)	4882 (5%)
**Socioeconomic**						
SVI	0.438	0.533	0.481	0.331	0.290	0.181
Median Income	691828	174093	611435	471923	692806	282019
Employed Labor	180853 (49%)	42157 (48%)	112030 (48%)	131042 (50%)	114350 (55%)	49440 (51%)
Unemployed Labor	11632 (3%)	2572 (3%)	5471 (2%)	6619 (3%)	3818 (2%)	1997 (2%)
**Healthcare Access**						
Total Hospital	15	1	4	3	4	3
Private	180243 (48%)	40439 (46%)	121450 (52%)	161626 (61%)	130119 (63%)	63159 (65%)
Medicare	14227 (4%)	4325 (5%)	10935 (5%)	12783 (5%)	12290 (6%)	5766 (6%)
Medicaid	50198 (14%)	12419 (14%)	30269 (13%)	27792 (11%)	13687 (7%)	3799 (4%)
Uninsured	32593 (9%)	8565 (10%)	22655 (10%)	21791 (8%)	14891 (7%)	3947 (4%)

PI* = Plum Island

MP* = Mount Pleasant

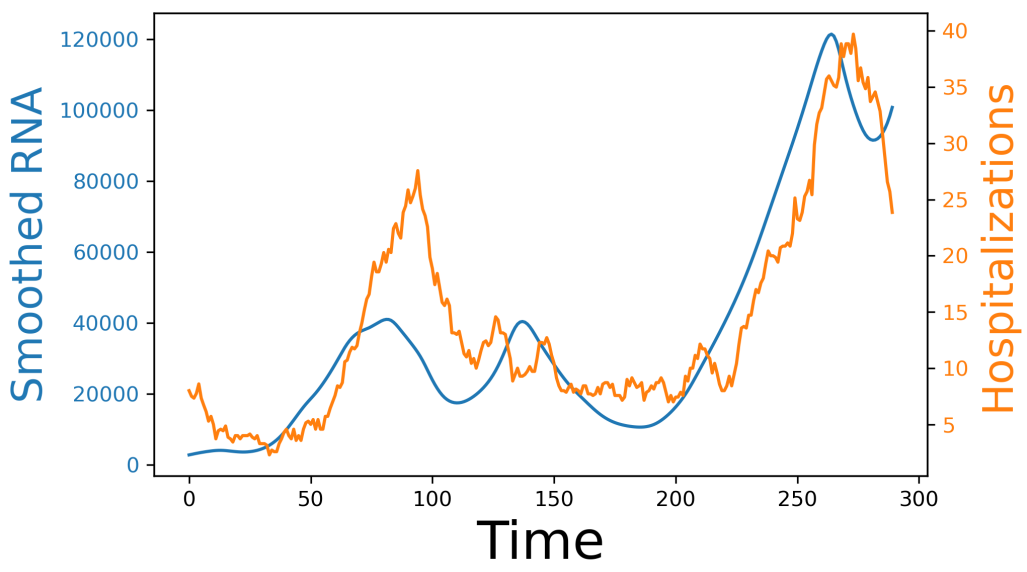

(a) Richland (Metro)

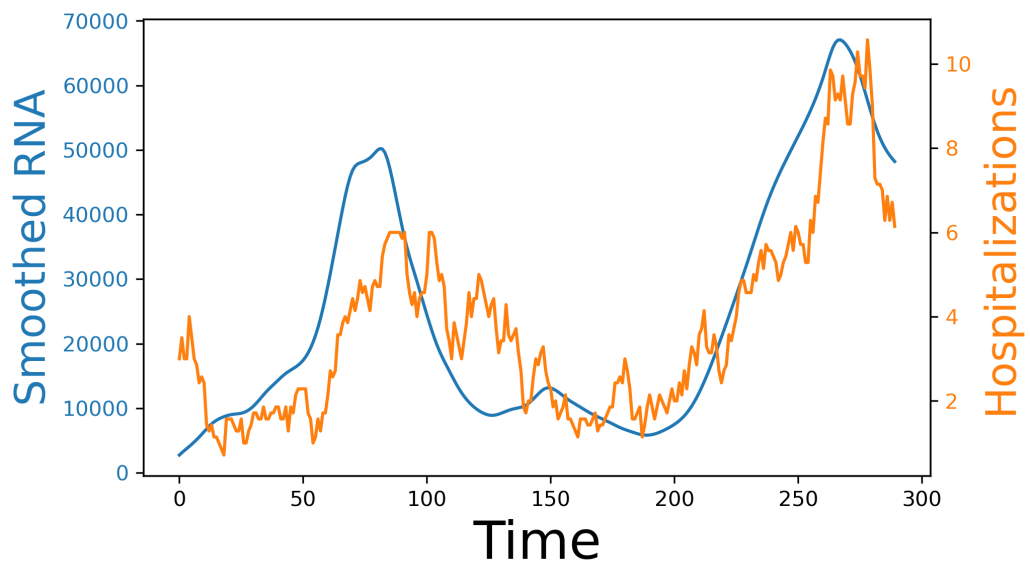

(b) Richaland (ER)

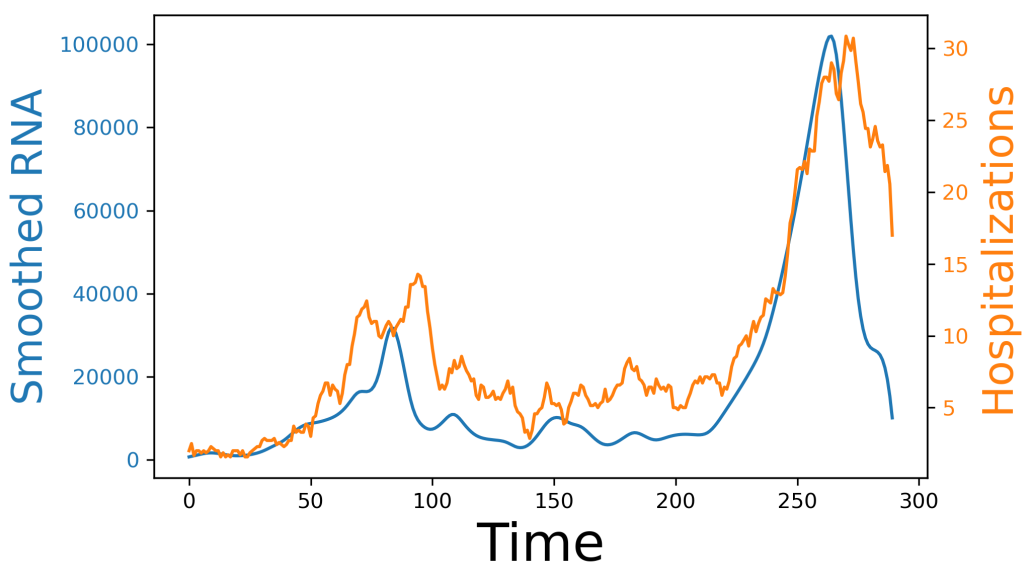

(c) Lexington

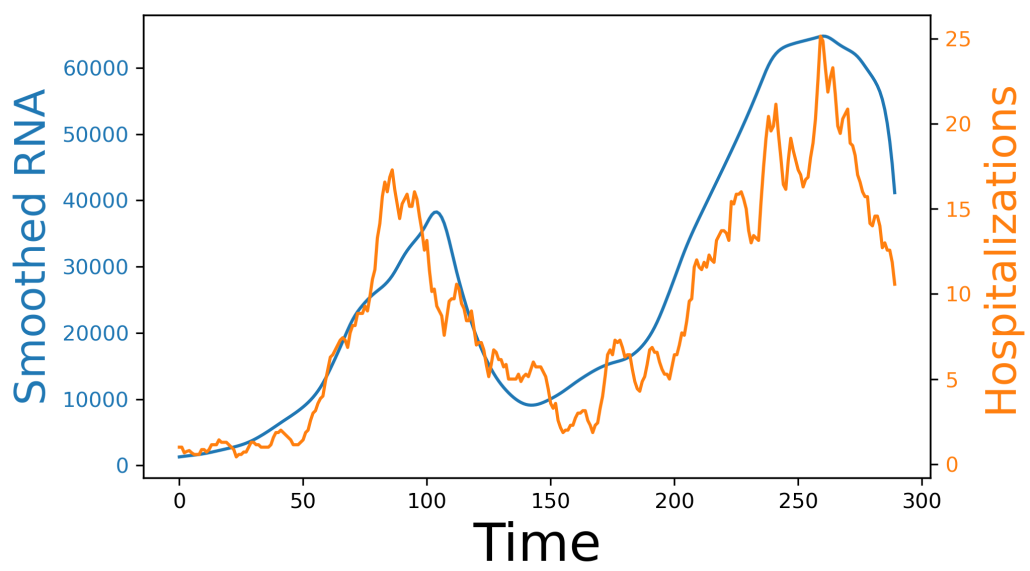

(d) York

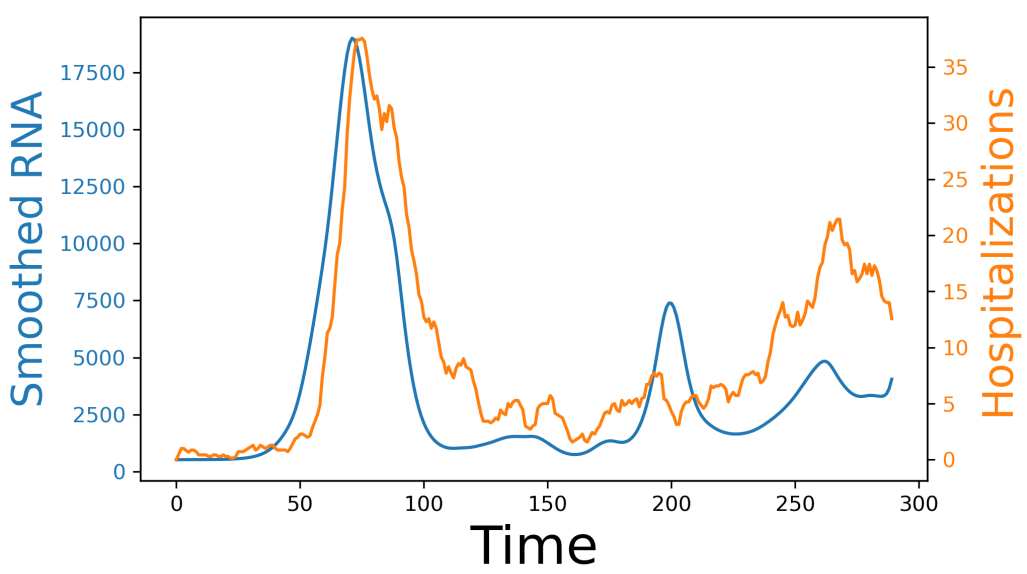

(e) Charleston (PI)

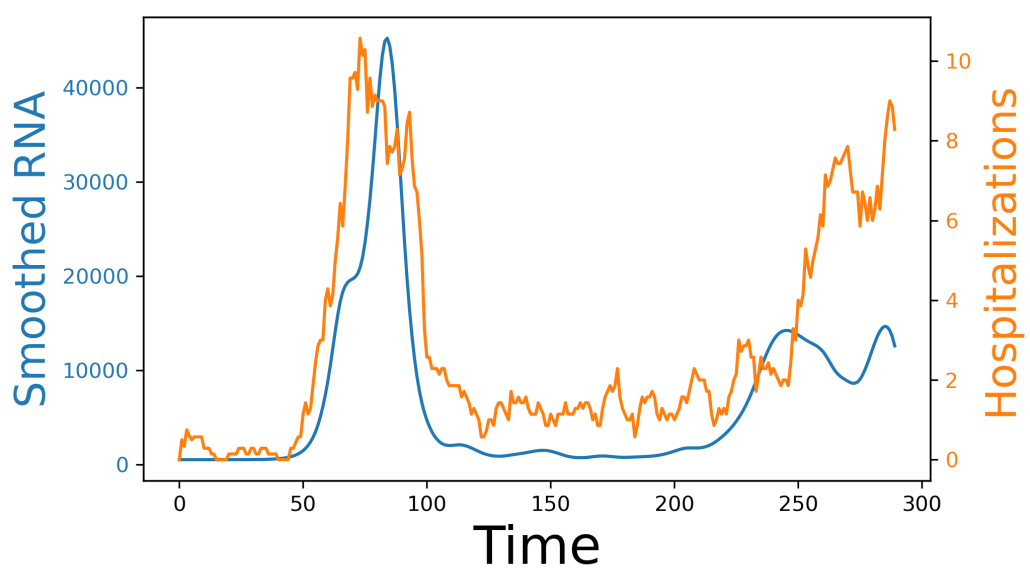

(f) Charleston (MP)

Figure A.1: Smoothed RNA Copy and Hospitalization Over Time for all the wastewater treatmant plants. The yellow and blue curves here denote the hospitalization and the smoothed RNA-copy respectively.

Additional accuracy metrics

Mean absolute error (MAE) measures the average absolute difference between observed and predicted values,

$$\mathrm{MAE} = \frac{1}{n}\sum_{t=1}^{n}|y_t - \hat{y}_t|.$$

Symmetric mean absolute percentage error(SMAPE) is a scale-free percentage error that symmetrically normalizes the absolute error by the sum of observed and predicted values,

$$\mathrm{sMAPE} = \frac{100}{n}\sum_{t=1}^{n}\frac{|y_t - \hat{y}_t|}{(|y_t| + |\hat{y}_t|)/2}.$$

Table A.2: Mean absolute error of predicted hospitalizations across all WWTP's, using COVID-19 RNA concentrations in wastewater for 7-, 14-, and 21-days ahead. Each model's performance was evaluated over a 28-day test period. The highest accuracy is highlighted.

Model	7-Day Ahead	14-Day Ahead	21-Day Ahead
Poisson Regression	29.89 (IQR: 14.98–38.76)	28.02 (IQR: 16.58–34.80)	41.99 (IQR: 21.56–59.98)
Random Forest	20.09 (IQR: 13.14-29.88)	**16.83 (IQR: 8.01–20.03)**	17.96 (IQR: 7.92–20.64)

Table A.3: Symmetric mean absolute percentage error of predicted hospitalizations across all WWTP's, using COVID-19 RNA concentrations in wastewater for 7-, 14-, and 21-days ahead. Each model's performance was evaluated over a 28-day test period. The highest accuracy is highlighted.

Model	7-Day Ahead	14-Day Ahead	21-Day Ahead
Poisson Regression	25.88 % (IQR: 23.01-33.32 %)	22.85 (IQR: 13.62–27.26)	30.62 (IQR: 22.79–39.56)
Random Forest	18.22 (IQR: 12.31-24.52)	**13.63 (IQR: 9.01–13.54)**	13.94 (IQR: 9.57–11.32)

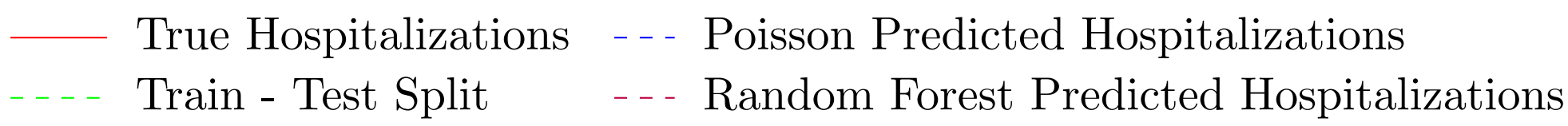

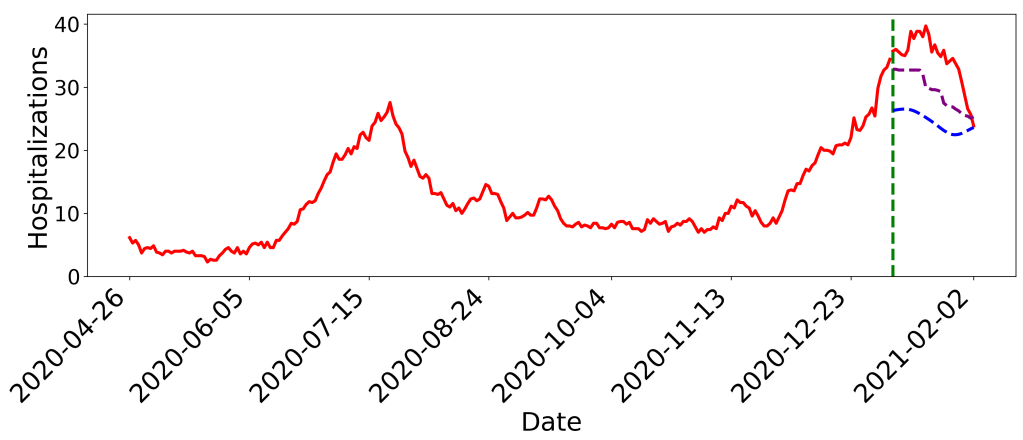

(a) Richland (Metro)

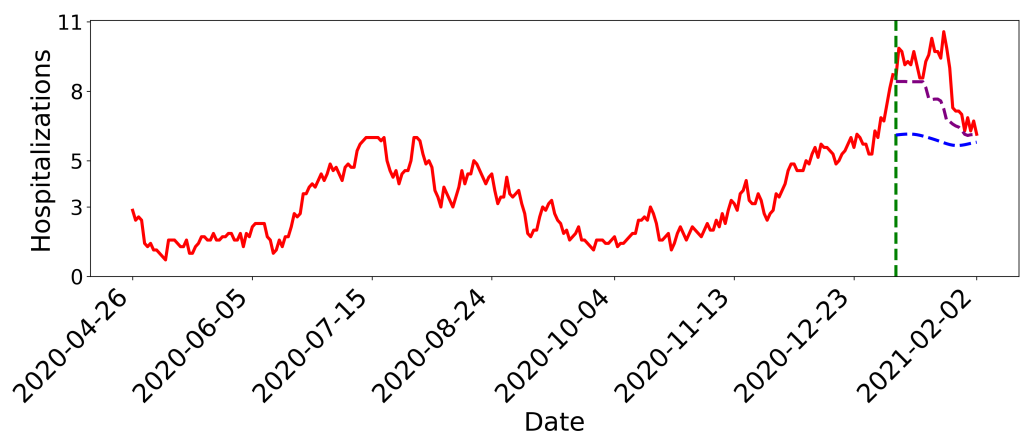

(b) Richaland (ER)

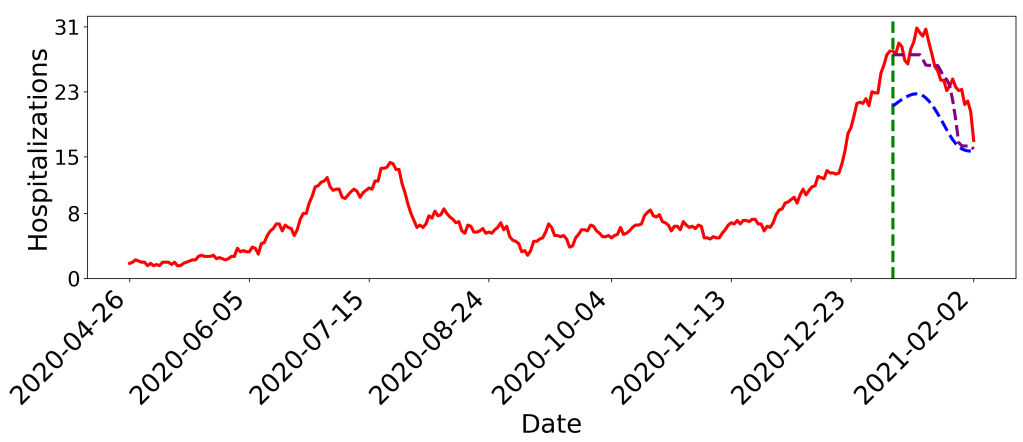

(c) Lexington

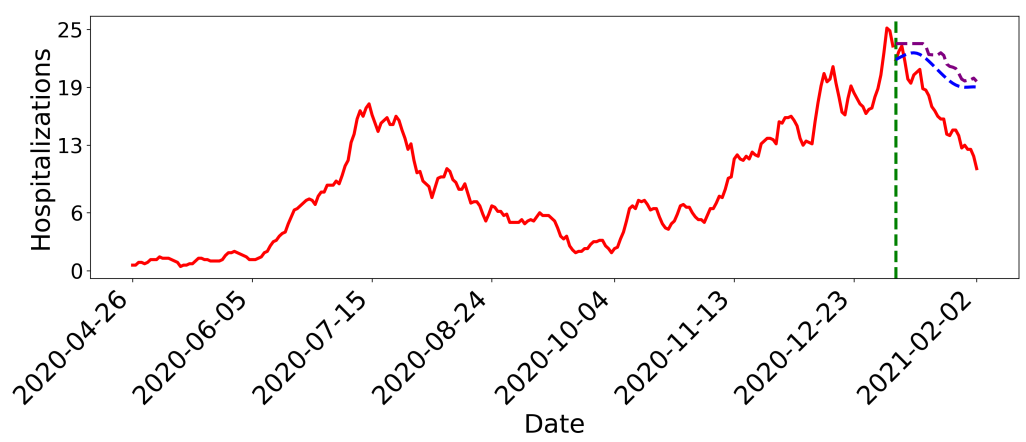

(d) York

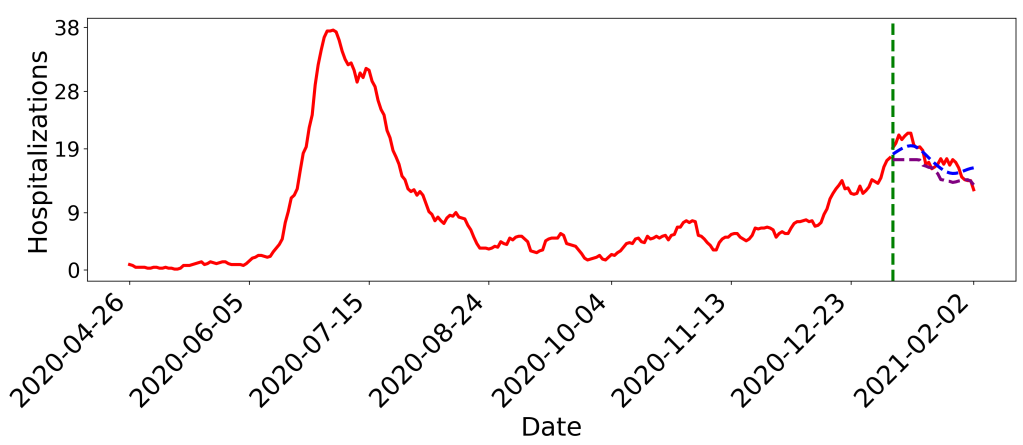

(e) Charleston (PI)

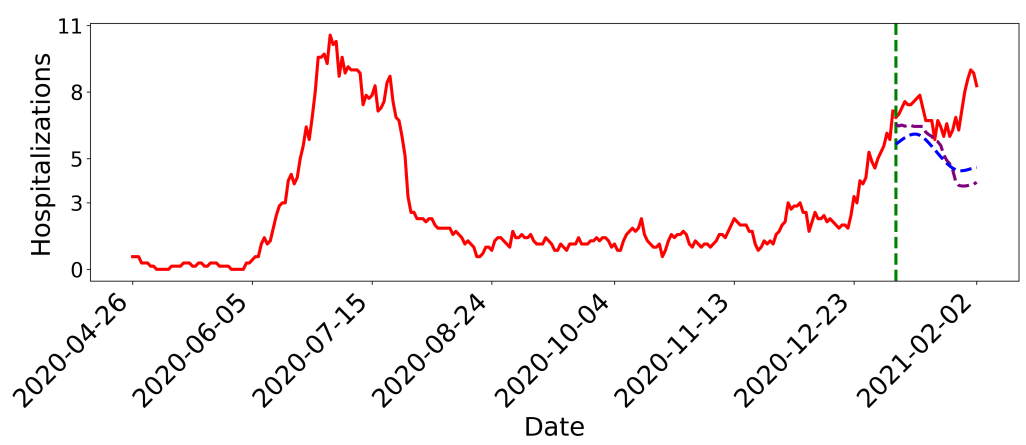

(f) Charleston (MP)

Figure A.2: Predicted and true COVID-19 hospitalizations across wastewater treatment plants (WWTPs) using 7-day ahead. The purple line represents predictions from the random forest model, the blue line shows predictions from the Poisson regression model, and the red line indicates true hospitalizations. The dashed green line marks the point dividing the training and testing periods.

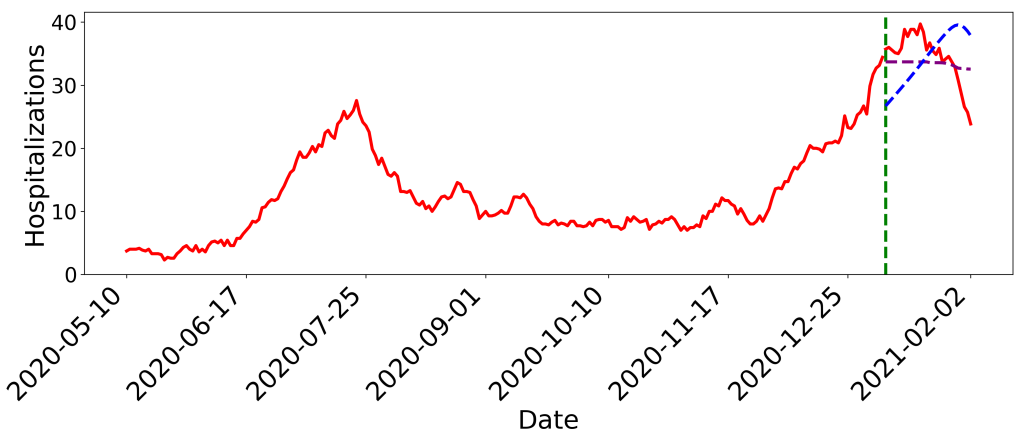

(a) Richland (Metro)

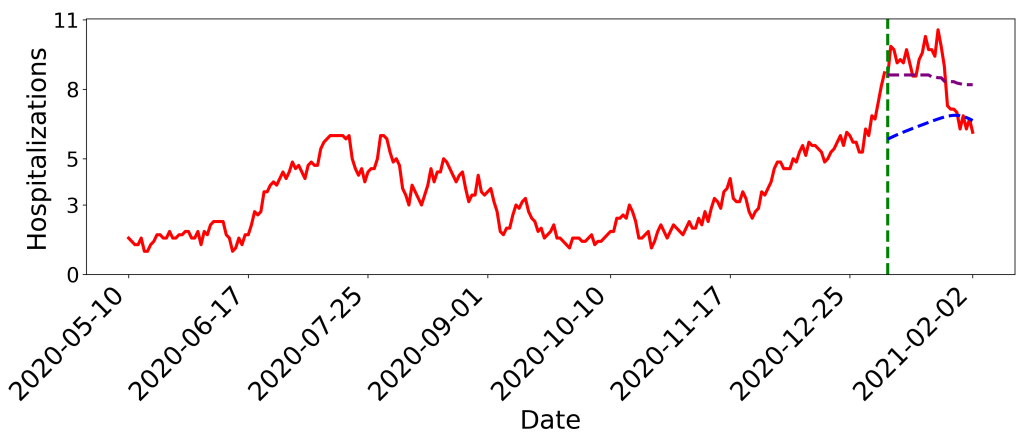

(b) Richaland (ER)

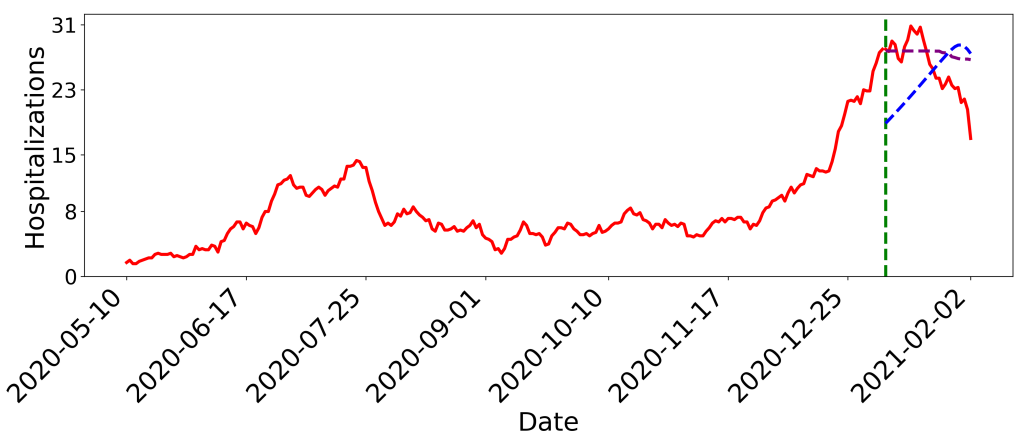

(c) Lexington

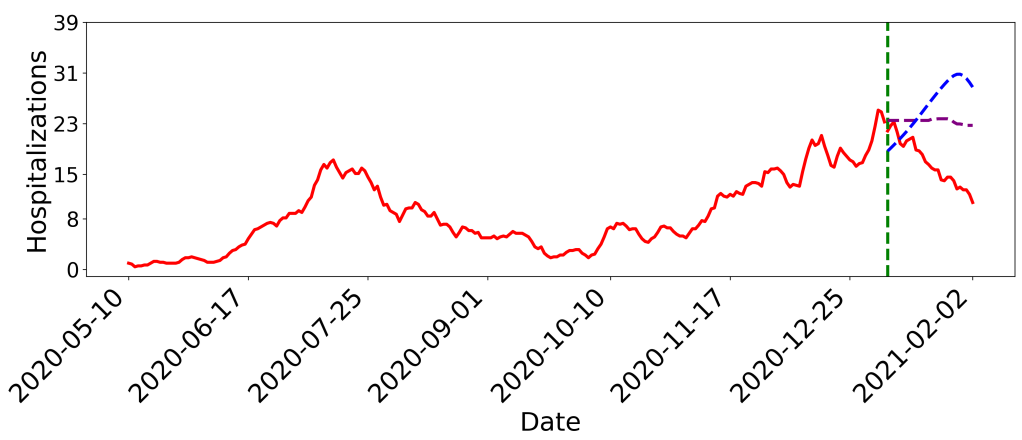

(d) York

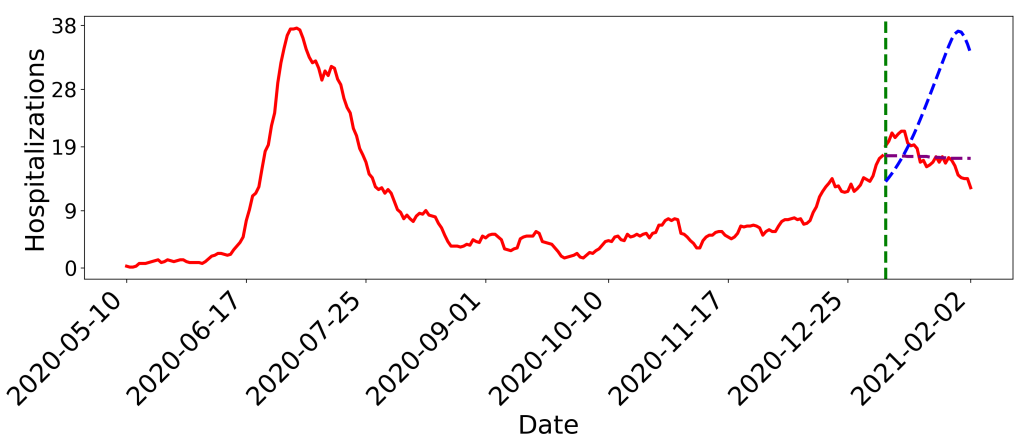

(e) Charleston (PI)

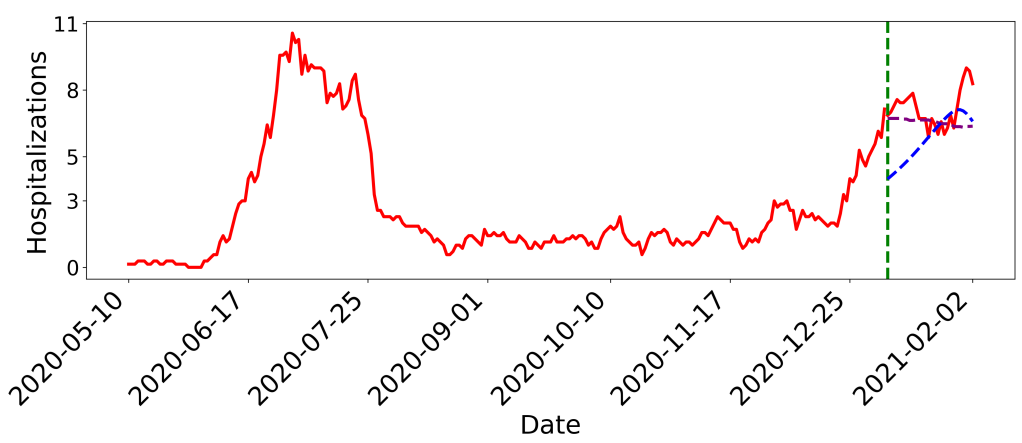

(f) Charleston (MP)

Figure A.3: Predicted and true COVID-19 hospitalizations across wastewater treatment plants (WWTPs) using 21-day ahead. The purple line represents predictions from the random forest model, the blue line shows predictions from the Poisson regression model, and the red line indicates true hospitalizations. The dashed green line marks the point dividing the training and testing periods.

Table A.4: Average Percentage Agreement over the prediction period Between Predicted and Observed COVID-19 Hospitalizations, Stratified by Modeling Approach, Days Ahead, and Wastewater Treatment Plant (WWTP) Service Area

WWTP	Model	Days Ahead	Average Percent Agreement
WWTP 1	Poisson Regression	5	67.55
WWTP 1	Poisson Regression	7	71.15
WWTP 1	Poisson Regression	14	90.20
WWTP 1	Poisson Regression	21	83.40
WWTP 1	Random Forest	5	83.51
WWTP 1	Random Forest	7	85.19
WWTP 1	Random Forest	14	91.60
WWTP 1	Random Forest	21	91.41
WWTP 2	Poisson Regression	5	68.01
WWTP 2	Poisson Regression	7	69.97
WWTP 2	Poisson Regression	14	76.59
WWTP 2	Poisson Regression	21	75.92
WWTP 2	Random Forest	5	83.39
WWTP 2	Random Forest	7	86.62
WWTP 2	Random Forest	14	90.41
WWTP 2	Random Forest	21	89.11

WWTP	Model	Days Ahead	Average Percent Agreement
WWTP 3	Poisson Regression	5	72.03
WWTP 3	Poisson Regression	7	76.32
WWTP 3	Poisson Regression	14	89.11
WWTP 3	Poisson Regression	21	80.04
WWTP 3	Random Forest	5	86.16
WWTP 3	Random Forest	7	91.70
WWTP 3	Random Forest	14	91.20
WWTP 3	Random Forest	21	90.58
WWTP 5	Poisson Regression	5	85.20
WWTP 5	Poisson Regression	7	81.05
WWTP 5	Poisson Regression	14	66.94
WWTP 5	Poisson Regression	21	66.96
WWTP 5	Random Forest	5	78.56
WWTP 5	Random Forest	7	76.56
WWTP 5	Random Forest	14	74.97
WWTP 5	Random Forest	21	72.50
WWTP 6	Poisson Regression	5	89.57
WWTP 6	Poisson Regression	7	94.98
WWTP 6	Poisson Regression	14	76.65
WWTP 6	Poisson Regression	21	63.32
WWTP 6	Random Forest	5	83.69
WWTP 6	Random Forest	7	89.16
WWTP 6	Random Forest	14	91.59
WWTP 6	Random Forest	21	91.14
WWTP 7	Poisson Regression	5	69.17
WWTP 7	Poisson Regression	7	73.44
WWTP 7	Poisson Regression	14	83.41
WWTP 7	Poisson Regression	21	80.23
WWTP 7	Random Forest	5	68.83
WWTP 7	Random Forest	7	75.13
WWTP 7	Random Forest	14	86.66
WWTP 7	Random Forest	21	90.79

B. ZIP code

Table B.5: Mean absolute error of predicted hospitalizations across all ZIP codes, using COVID-19 RNA concentrations in wastewater for 7-, 14-, and 21-days ahead. Each model's performance was evaluated over a 28-day test period. The highest accuracy is highlighted.

Model	7-Day Ahead	14-Day Ahead	21-Day Ahead
Poisson Regression	5.05 (IQR: 2.7–7.22)	6.21 (IQR: 2.86–7.67)	8.01 (IQR: 3.38–9.53)
Random Forest	4.97 (IQR: 3.57-5.91)	**4.74 (IQR: 2.63–5.55)**	5.05 (IQR: 2.77–5.74)

Table B.6: Symmetric mean absolute percentage error of predicted hospitalizations across all ZIP codes, using COVID-19 RNA concentrations in wastewater for 7-, 14-, and 21-days ahead. Each model's performance was evaluated over a 28-day test period. The highest accuracy is highlighted.

Model	7-Day Ahead	14-Day Ahead	21-Day Ahead
Poisson Regression	33.95 % (IQR: 20.73-43.65 %)	35.70 (IQR: 22.78–42.03)	42.46 (IQR: 28.76–39.07)
Random Forest	34.68 (IQR: 17.19-43.33)	**32.17 (IQR: 17.83–41.04)**	32.95 (IQR: 18.19–42.77)

Table B.7: Average Percentage Agreement in the Prediction Period Between Predicted and Observed COVID-19 Hospitalizations, Stratified by Modeling Approach, Days Ahead, and ZIP code

ZIP code	Model	Days Ahead	Average Percentage Agreement
29016	Poisson Regression	5	82.55
29016	Poisson Regression	7	85.51
29016	Poisson Regression	14	80.57
29016	Poisson Regression	21	76.23
29016	Random Forest	5	85.12
29016	Random Forest	7	82.80
29016	Random Forest	14	79.86
29016	Random Forest	21	78.20
29033	Poisson Regression	5	75.98
29033	Poisson Regression	7	79.99
29033	Poisson Regression	14	83.33
29033	Poisson Regression	21	87.73
29033	Random Forest	5	81.16
29033	Random Forest	7	81.09
29033	Random Forest	14	85.34
29033	Random Forest	21	85.07
29053	Poisson Regression	5	61.67
29053	Poisson Regression	7	65.29
29053	Poisson Regression	14	76.62
29053	Poisson Regression	21	71.97
29053	Random Forest	5	58.20
29053	Random Forest	7	85.17
29053	Random Forest	14	87.68
29053	Random Forest	21	85.85
29054	Poisson Regression	5	62.16
29054	Poisson Regression	7	65.32
29054	Poisson Regression	14	68.61
29054	Poisson Regression	21	64.05
29054	Random Forest	5	68.02
29054	Random Forest	7	64.15
29054	Random Forest	14	67.57
29054	Random Forest	21	62.79
29063	Poisson Regression	5	85.40
29063	Poisson Regression	7	87.41
29063	Poisson Regression	14	81.58
29063	Poisson Regression	21	75.40
29063	Random Forest	5	86.05
29063	Random Forest	7	90.28
29063	Random Forest	14	91.34
29063	Random Forest	21	90.49

ZIP code	Model	Days Ahead	Average Percentage Agreement
29070	Poisson Regression	5	66.32
29070	Poisson Regression	7	72.16
29070	Poisson Regression	14	86.70
29070	Poisson Regression	21	77.05
29070	Random Forest	5	81.21
29070	Random Forest	7	85.78
29070	Random Forest	14	84.91
29070	Random Forest	21	84.87
29072	Poisson Regression	5	88.17
29072	Poisson Regression	7	91.91
29072	Poisson Regression	14	82.32
29072	Poisson Regression	21	76.45
29072	Random Forest	5	90.77
29072	Random Forest	7	90.75
29072	Random Forest	14	84.70
29072	Random Forest	21	83.21
29073	Poisson Regression	5	74.92
29073	Poisson Regression	7	77.02
29073	Poisson Regression	14	75.14
29073	Poisson Regression	21	69.80
29073	Random Forest	5	79.48
29073	Random Forest	7	78.95
29073	Random Forest	14	76.82
29073	Random Forest	21	75.92
29160	Poisson Regression	5	61.70
29160	Poisson Regression	7	60.04
29160	Poisson Regression	14	61.39
29160	Poisson Regression	21	66.56
29160	Random Forest	5	71.04
29160	Random Forest	7	73.13
29160	Random Forest	14	70.49
29160	Random Forest	21	72.37
29169	Poisson Regression	5	59.84
29169	Poisson Regression	7	63.62
29169	Poisson Regression	14	66.01
29169	Poisson Regression	21	52.63
29169	Random Forest	5	63.44
29169	Random Forest	7	65.71
29169	Random Forest	14	78.27
29169	Random Forest	21	80.85

ZIP code	Model	Days Ahead	Average Percentage Agreement
29170	Poisson Regression	5	66.93
29170	Poisson Regression	7	70.07
29170	Poisson Regression	14	77.93
29170	Poisson Regression	21	74.78
29170	Random Forest	5	68.82
29170	Random Forest	7	74.75
29170	Random Forest	14	82.48
29170	Random Forest	21	78.43
29172	Poisson Regression	5	67.74
29172	Poisson Regression	7	68.32
29172	Poisson Regression	14	73.40
29172	Poisson Regression	21	68.62
29172	Random Forest	5	54.69
29172	Random Forest	7	63.32
29172	Random Forest	14	68.45
29172	Random Forest	21	66.17
29201	Poisson Regression	5	60.75
29201	Poisson Regression	7	62.69
29201	Poisson Regression	14	70.53
29201	Poisson Regression	21	70.05
29201	Random Forest	5	63.17
29201	Random Forest	7	64.67
29201	Random Forest	14	71.98
29201	Random Forest	21	70.58
29203	Poisson Regression	5	64.98
29203	Poisson Regression	7	69.13
29203	Poisson Regression	14	93.62
29203	Poisson Regression	21	82.06
29203	Random Forest	5	86.93
29203	Random Forest	7	88.53
29203	Random Forest	14	92.39
29203	Random Forest	21	92.28
29204	Poisson Regression	5	51.55
29204	Poisson Regression	7	52.63
29204	Poisson Regression	14	57.77
29204	Poisson Regression	21	57.76
29204	Random Forest	5	74.62
29204	Random Forest	7	79.36
29204	Random Forest	14	89.46
29204	Random Forest	21	86.11

ZIP code	Model	Days Ahead	Average Percentage Agreement
29205	Poisson Regression	5	64.84
29205	Poisson Regression	7	67.64
29205	Poisson Regression	14	68.26
29205	Poisson Regression	21	63.64
29205	Random Forest	5	64.35
29205	Random Forest	7	73.93
29205	Random Forest	14	64.51
29205	Random Forest	21	63.49
29206	Poisson Regression	5	71.30
29206	Poisson Regression	7	73.16
29206	Poisson Regression	14	74.33
29206	Poisson Regression	21	66.00
29206	Random Forest	5	80.16
29206	Random Forest	7	77.87
29206	Random Forest	14	76.70
29206	Random Forest	21	75.24
29209	Poisson Regression	5	81.15
29209	Poisson Regression	7	83.13
29209	Poisson Regression	14	79.54
29209	Poisson Regression	21	70.38
29209	Random Forest	5	80.98
29209	Random Forest	7	80.38
29209	Random Forest	14	75.61
29209	Random Forest	21	72.01
29210	Poisson Regression	5	58.99
29210	Poisson Regression	7	60.92
29210	Poisson Regression	14	71.39
29210	Poisson Regression	21	80.41
29210	Random Forest	5	40.98
29210	Random Forest	7	39.62
29210	Random Forest	14	82.59
29210	Random Forest	21	72.42
29212	Poisson Regression	5	85.21
29212	Poisson Regression	7	91.19
29212	Poisson Regression	14	88.71
29212	Poisson Regression	21	78.11
29212	Random Forest	5	87.46
29212	Random Forest	7	88.22
29212	Random Forest	14	88.77
29212	Random Forest	21	88.70

ZIP code	Model	Days Ahead	Average Percentage Agreement
29223	Poisson Regression	5	76.28
29223	Poisson Regression	7	77.82
29223	Poisson Regression	14	87.89
29223	Poisson Regression	21	80.71
29223	Random Forest	5	85.80
29223	Random Forest	7	86.16
29223	Random Forest	14	84.80
29223	Random Forest	21	87.73
29401	Poisson Regression	5	48.84
29401	Poisson Regression	7	49.72
29401	Poisson Regression	14	49.40
29401	Poisson Regression	21	46.21
29401	Random Forest	5	30.22
29401	Random Forest	7	33.34
29401	Random Forest	14	32.02
29401	Random Forest	21	44.82
29403	Poisson Regression	5	54.48
29403	Poisson Regression	7	58.14
29403	Poisson Regression	14	72.64
29403	Poisson Regression	21	75.34
29403	Random Forest	5	44.20
29403	Random Forest	7	49.88
29403	Random Forest	14	51.78
29403	Random Forest	21	50.88
29407	Poisson Regression	5	88.95
29407	Poisson Regression	7	85.64
29407	Poisson Regression	14	72.24
29407	Poisson Regression	21	54.63
29407	Random Forest	5	86.57
29407	Random Forest	7	84.04
29407	Random Forest	14	83.17
29407	Random Forest	21	80.69
29412	Poisson Regression	5	74.60
29412	Poisson Regression	7	72.95
29412	Poisson Regression	14	66.75
29412	Poisson Regression	21	64.52
29412	Random Forest	5	66.34
29412	Random Forest	7	76.60
29412	Random Forest	14	77.27
29412	Random Forest	21	76.69
29414	Poisson Regression	5	86.52
29414	Poisson Regression	7	82.96
29414	Poisson Regression	14	70.44
29414	Poisson Regression	21	61.86

ZIP code	Model	Days Ahead	Average Percentage Agreement
29414	Random Forest	5	84.33
29414	Random Forest	7	85.89
29414	Random Forest	14	86.82
29414	Random Forest	21	85.28
29429	Poisson Regression	5	58.20
29429	Poisson Regression	7	59.28
29429	Poisson Regression	14	53.19
29429	Poisson Regression	21	39.45
29429	Random Forest	5	27.91
29429	Random Forest	7	23.57
29429	Random Forest	14	28.46
29429	Random Forest	21	33.36
29449	Poisson Regression	5	83.57
29449	Poisson Regression	7	84.07
29449	Poisson Regression	14	71.55
29449	Poisson Regression	21	62.40
29449	Random Forest	5	74.30
29449	Random Forest	7	85.15
29449	Random Forest	14	79.49
29449	Random Forest	21	85.88
29455	Poisson Regression	5	74.86
29455	Poisson Regression	7	78.28
29455	Poisson Regression	14	76.53
29455	Poisson Regression	21	72.83
29455	Random Forest	5	63.10
29455	Random Forest	7	65.44
29455	Random Forest	14	65.44
29455	Random Forest	21	64.92
29464	Poisson Regression	5	88.83
29464	Poisson Regression	7	91.37
29464	Poisson Regression	14	70.01
29464	Poisson Regression	21	63.34
29464	Random Forest	5	53.95
29464	Random Forest	7	71.88
29464	Random Forest	14	84.30
29464	Random Forest	21	83.38
29466	Poisson Regression	5	67.83
29466	Poisson Regression	7	75.82
29466	Poisson Regression	14	81.53
29466	Poisson Regression	21	84.48
29466	Random Forest	5	55.13
29466	Random Forest	7	68.35
29466	Random Forest	14	82.50
29466	Random Forest	21	87.84

ZIP code	Model	Days Ahead	Average Percentage Agreement
29470	Poisson Regression	5	68.78
29470	Poisson Regression	7	66.37
29470	Poisson Regression	14	65.10
29470	Poisson Regression	21	73.62
29470	Random Forest	5	88.29
29470	Random Forest	7	81.85
29470	Random Forest	14	77.17
29470	Random Forest	21	77.29
29492	Poisson Regression	5	82.74
29492	Poisson Regression	7	78.52
29492	Poisson Regression	14	62.67
29492	Poisson Regression	21	53.42
29492	Random Forest	5	87.30
29492	Random Forest	7	77.00
29492	Random Forest	14	63.62
29492	Random Forest	21	59.10
29708	Poisson Regression	5	72.83
29708	Poisson Regression	7	74.44
29708	Poisson Regression	14	81.85
29708	Poisson Regression	21	74.91
29708	Random Forest	5	64.44
29708	Random Forest	7	71.91
29708	Random Forest	14	67.76
29708	Random Forest	21	66.32
29710	Poisson Regression	5	91.29
29710	Poisson Regression	7	90.47
29710	Poisson Regression	14	79.82
29710	Poisson Regression	21	78.14
29710	Random Forest	5	86.91
29710	Random Forest	7	85.62
29710	Random Forest	14	84.21
29710	Random Forest	21	82.46
29715	Poisson Regression	5	58.38
29715	Poisson Regression	7	56.93
29715	Poisson Regression	14	50.38
29715	Poisson Regression	21	50.79
29715	Random Forest	5	66.95
29715	Random Forest	7	62.43
29715	Random Forest	14	65.23
29715	Random Forest	21	68.54
29730	Poisson Regression	5	74.07
29730	Poisson Regression	7	69.76
29730	Poisson Regression	14	50.07
29730	Poisson Regression	21	51.66

ZIP code	Model	Days Ahead	Average Percentage Agreement
29730	Random Forest	5	70.70
29730	Random Forest	7	68.83
29730	Random Forest	14	65.85
29730	Random Forest	21	65.20
29732	Poisson Regression	5	79.96
29732	Poisson Regression	7	81.28
29732	Poisson Regression	14	74.07
29732	Poisson Regression	21	68.26
29732	Random Forest	5	81.16
29732	Random Forest	7	78.21
29732	Random Forest	14	72.40
29732	Random Forest	21	70.50
29745	Poisson Regression	5	73.02
29745	Poisson Regression	7	69.24
29745	Poisson Regression	14	53.95
29745	Poisson Regression	21	55.42
29745	Random Forest	5	62.55
29745	Random Forest	7	64.39
29745	Random Forest	14	61.28
29745	Random Forest	21	58.75
